# A Portable Electrochemical Analysis System Integrated with Machine Learning for Rapid Detection of Pungency Intensity in Red and Green Szechuan Peppers (*Zanthoxylum bungeanum* and *Zanthoxylum schinifolium*)

**DOI:** 10.3390/foods15172948

**Published:** 2026-08-22

**Authors:** Di Zhang, Bin Zhang, Shiyu Huang, Xiaobo Zou, Zitao Lin, Kui Zhong, Lei Zhao, Bolin Shi, Lingqin Shen

**Affiliations:** 1 Key Laboratory of Food Sensory Analysis, State Administration for Market Regulation, Beijing 100191, China; d.zhang@ujs.edu.cn (D.Z.); zhongkui@cnis.ac.cn (K.Z.); shibl@cnis.ac.cn (B.S.); 2 School of Food and Biological Engineering, Jiangsu University, Zhenjiang 212013, China; 19741709336@163.com (B.Z.); hsy15855912106@163.com (S.H.); zou_xiaobo@ujs.edu.cn (X.Z.); 3 Jinan Fruit Research Institute, China Federation of Supply and Marketing Co-Operatives, Jinan 250200, China; 715198511@163.com; 4 Institute of Agri-Food Standardization, China National Institute of Standardization, Beijing 100191, China; 5 School of Chemistry and Chemical Engineering, Jiangsu University, Zhenjiang 212013, China

**Keywords:** electrochemical fingerprinting, Szechuan pepper, numbing intensity, differential pulse voltammetry, pungency, standard normal variate, machine learning

## Abstract

Szechuan pepper pungency is traditionally assessed by subjective sensory panels or laboratory instruments, hindering on-site detection. Perceived numbing sensation does not always correspond directly to alkylamide content because multiple electroactive constituents, including polyphenols and sanshools, may contribute to the overall sensory response. Chromatographic methods quantify individual compounds but may not fully reflect integrated human pungency perception. Electrochemical detection bypasses separation, as the voltammetric response integrates oxidative signals from multiple electroactive species. We developed a portable electrochemical system with a custom programmable-gain potentiostat, three-electrode detector, and STM32-controlled software for differential pulse voltammetry (DPV) measurement. Coupled with machine learning, it assessed Zanthoxylum bungeanum (red peppers) and Zanthoxylum schinifolium (green peppers). Fifteen replicate scans from each of 22 origins yielded 330 DPV curves calibrated against general Labeled Magnitude Scale (gLMS) scores from a trained panel. An artificial neural network (ANN) achieved R^2^ = 0.937 for red peppers, while principal component analysis–support vector regression (PCA–SVR) achieved R^2^ = 0.860 for green peppers. Competitive adaptive reweighted sampling (CARS) identified three characteristic potential intervals for each type: 0.17–0.21, 0.57–0.62, and 0.69–0.80 V for red peppers, and 0.24–0.27, 0.56–0.68, and 0.69–0.77 V for green peppers. These intervals indicate that pungency-related electrochemical information is distributed across multiple potential regions. The system shows potential for rapid and objective quality assessment of Szechuan pepper.

## 1. Introduction

Szechuan pepper is valued for a distinctive chemesthetic sensation characterized by tingling and numbness and is also an important economic crop in China [1,2]. Reliable assessment of this sensation is relevant to raw-material grading, product formulation, and industrial quality control. In the present study, the term “pungency” is used only in the broad sense adopted in parts of the Szechuan pepper literature, whereas the specific sensory endpoint quantified by the general Labeled Magnitude Scale (gLMS) and predicted by the proposed system is numbing intensity. This hydroxy-sanshool-mediated tingling/numbing response is distinct from the capsaicinoid-mediated burning pungency of *Capsicum* peppers. Trained sensory panels directly reflect human perception, but assessor differences, fatigue, adaptation, sample order, and environmental conditions can limit reproducibility and throughput [3,4].

Gas chromatography, liquid chromatography, and near-infrared spectroscopy have been used to characterize numbing-related constituents [5,6,7]. Chromatographic methods quantify alkylamides, particularly hydroxyl-sanshools, with high chemical specificity; however, perceived intensity does not always correlate strictly with total alkylamide concentration [8,9]. Relative proportions of hydroxy-α-, hydroxy-β-, hydroxy-γ-sanshool and related unsaturated amides, oxidative degradation, extraction behavior, and interactions with other matrix constituents may all influence the integrated sensation. Conventional instruments also generally require expensive, bulky equipment, specialized operators, and time-consuming extraction, separation, and interpretation [10,11]. These constraints limit direct use for field screening and production-line-adjacent quality assessment.

Electrochemical analysis offers a potential alternative because ethanol extracts of Szechuan pepper contain several electroactive constituents, including alkylamides and phenolic compounds [12,13,14,15,16,17,18]. Phenolic constituents may also be relevant to the sensory endpoint rather than being merely electroactive background components. Certain polyphenols have been reported to activate human TRPA1 and TRPV1 channels involved in oral chemesthetic signaling [19], providing a plausible biological basis for their potential influence on the integrated sensory response. A differential pulse voltammetry (DPV) trace from an unseparated extract therefore represents a cross-reactive electrochemical fingerprint containing contributions from multiple coextracted constituents. Because structurally related hydroxyl-sanshools can produce closely spaced and partially overlapping oxidation responses, while phenolic constituents also contribute to the electrochemical profile, an individual current response cannot be assigned selectively to a single compound without compound-specific standards and appropriate validation. Accordingly, the present work relates the integrated DPV fingerprint directly to sensory numbing intensity rather than treating the device as a molecule-specific detector.

Previous electrochemical studies quantified individual sanshools using benchtop workstations and related DPV fingerprints to geographical origin and sensory intensity [15,20]. Nevertheless, an integrated portable hardware–software system capable of mapping whole-extract DPV fingerprints directly to trained-panel gLMS scores has not been established. The absence of a compact analyzer that combines programmable potential control, weak-current acquisition, reproducible preprocessing, and automated sensory-score inference therefore represents the principal technological gap addressed in this work.

Red Szechuan pepper (*Zanthoxylum bungeanum*) and green Szechuan pepper (*Zanthoxylum schinifolium*) differ in botanical origin, alkylamide composition, aroma, and sensory properties [21,22]. Separate descriptive and predictive analyses were therefore retained for the two types. We hypothesized that their cross-reactive DPV fingerprints contain integrated information associated with sensory numbing intensity, while recognizing that geographical origin, maturity, drying history, and other matrix factors may also contribute.

This study developed a portable battery-powered electrochemical fingerprinting system integrating a programmable-gain potentiostat, a three-electrode detector, an STM32 control unit, and mobile/cloud or local-computer inference [23,24,25,26]. DPV fingerprints were related to gLMS sensory scores; linear and nonlinear regressors were compared; competitive adaptive reweighted sampling (CARS) was used to locate predictive potential regions; and SHapley Additive exPlanations (SHAP) quantified the roles of peak current and other electrochemical descriptors. The resulting system is intended to support rapid on-site quality control, raw-material grading, and supply-chain screening of Szechuan pepper.

## 2. Materials and Methods

### 2.1. Chemicals and Materials

Purified hydroxy-α-sanshool (α-SOH, purity ≥ 98%, Chengdu RefMedic Biotech Co., Ltd., Chengdu, China) was used as the reference standard. Food-grade ethanol was purchased from Binzhou Guanglian Biochemical Co., Ltd. (Binzhou, China). Alumina powder (0.5 μm) was purchased from Baird Material Technology Co., Ltd. (Suzhou, China). Phosphate-buffered saline (PBS; 0.10 M, pH 5.6) was prepared with analytical-grade reagents and ultrapure water. The three-electrode system comprised a glassy-carbon working electrode, an Ag/AgCl reference electrode, and a platinum-wire counter electrode (CH Instruments Co., Ltd., Shanghai, China).

### 2.2. Sample Collection and Preparation

#### 2.2.1. Collection of Samples

Commercial harvesting of Sichuan pepper varies among pepper types, cultivars, production regions, and production seasons. In major production areas, the harvest period may range from late May or June for some green Szechuan peppers to July–September for many red Szechuan peppers and peppers grown in relatively cool or high-altitude regions. Because exact harvest dates are also affected by local climatic conditions, calendar date alone was not used to define maturity in this study.

Twenty-two commercially mature origin groups from the 2025 production season were evaluated: 15 red Z. bungeanum groups and 7 green Z. schinifolium groups. All samples were sourced during the normal local commercial harvest period and were required to meet type-specific commercial-maturity criteria. Zanthoxylum bungeanum (red pepper) samples had well-developed, fully expanded red-to-purple-red pericarps, whereas *Zanthoxylum schinifolium* (green pepper) samples had well-developed, fully expanded green-to-green-brown pericarps. Both types were required to exhibit prominent oil glands; visibly immature, markedly shriveled, mold-damaged, or insect-damaged material was excluded. The red groups were Wudu, Qinan, Gangu, Yuanlong, Danba, Linxia, Maoxian, Wenchuan, Hanyuan, Yanyuan, Fuping, Shexian, Laiwu (small granule), Laiwu (large granule), and Yuncheng. The green groups were Yongshan, Yiliang, Lugao, Ludian, Xianfeng, Yantan, and Jialing.

#### 2.2.2. Preparation of Samples

The samples were obtained as commercially dried pericarps; therefore, no additional drying treatment was performed in the laboratory before grinding. According to the Chinese national standard GB/T 30391-2024 Huajiao Pepper (Szechuan Pepper) [27], Szechuan pepper is the dried pericarp of *Zanthoxylum* species and its moisture mass fraction should not exceed 12.0%. The dried pericarps were ground and passed through a 20-mesh sieve. Ground material (2.00 g) was transferred to a 25 mL amber conical flask, 15 mL of anhydrous ethanol was added, and the mixture was shaken and sonicated at room temperature for 20 min [28,29]. After centrifugation at 2000 rpm for 10 min, the supernatant was transferred to a 20 mL amber volumetric flask, brought to volume with ethanol, and stored at −18 °C until electrochemical analysis.

### 2.3. System Design

#### 2.3.1. System Overview

**Figure 1:**
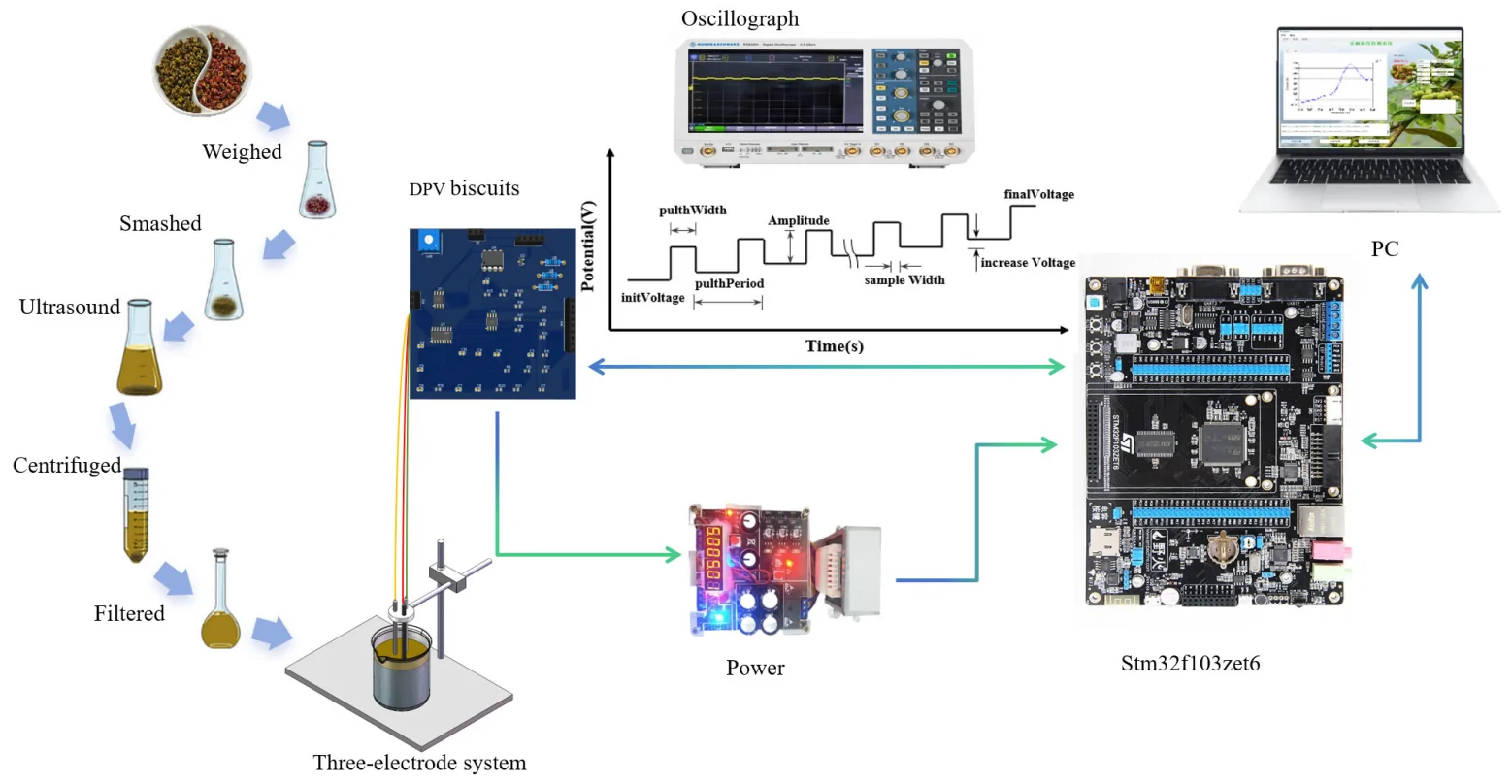
Schematic of the portable electrochemical fingerprinting workflow and prototype hardware for estimating Szechuan pepper numbing intensity.

The system comprises a three-electrode cell, a compact electrochemical controller, and an interchangeable computation interface (Figure 1). The controller integrates the main board, regulated power circuitry, analog front end, wireless communication module, and rechargeable battery in an enclosure of approximately 15 cm × 10 cm × 5 cm with a mass of approximately 0.5 kg [25,30]. In field or mobile use, the analyzer can operate from its battery and transmit the fingerprint to a mobile gateway for cloud inference. In factory or laboratory settings, it can instead use adapter power and a model deployed locally on a laptop or desktop computer.

The controller generated the DPV waveform from −0.20 to 1.20 V, yielding 342 potential–current pairs at a mean potential increment of approximately 4.1 mV. The pulse amplitude was 50 mV, pulse width 50 ms, sampling window 16.7 ms, pulse period 0.4 s, and quiet time 2 s. One complete fingerprint acquisition required approximately 2 min. Potential–current pairs were transferred by Bluetooth or Wi-Fi and cached locally if a network connection was temporarily unavailable.

#### 2.3.2. Selection and Application of Electronic Components

The controller is based on an STM32F103ZET6 microcontroller (STMicroelectronics, Geneva, Switzerland), which provides three analog-to-digital converters and two digital-to-analog converters. The PF0–PF2 pins control an ADG408BRZ eight-channel analog multiplexer (Analog Devices, Inc., Wilmington, MA, USA), which selects the feedback resistor used to extend the measurable current range. The nominal sensitivity settings and current ranges are listed in Table 1.

The electronics require regulated 3.3 V for the microcontroller and isolated ±5 V rails for the analog circuitry. In the portable configuration, these rails are supplied by a 12 V, 5 Ah rechargeable lithium-ion battery through isolated DC–DC conversion. At the measured average system load, one charge supports approximately 8 h of intermittent operation. A 220 V adapter is used for charging or continuous bench operation and is not required during field measurements.

#### 2.3.3. Peripheral Circuit Design

**Figure 2:**
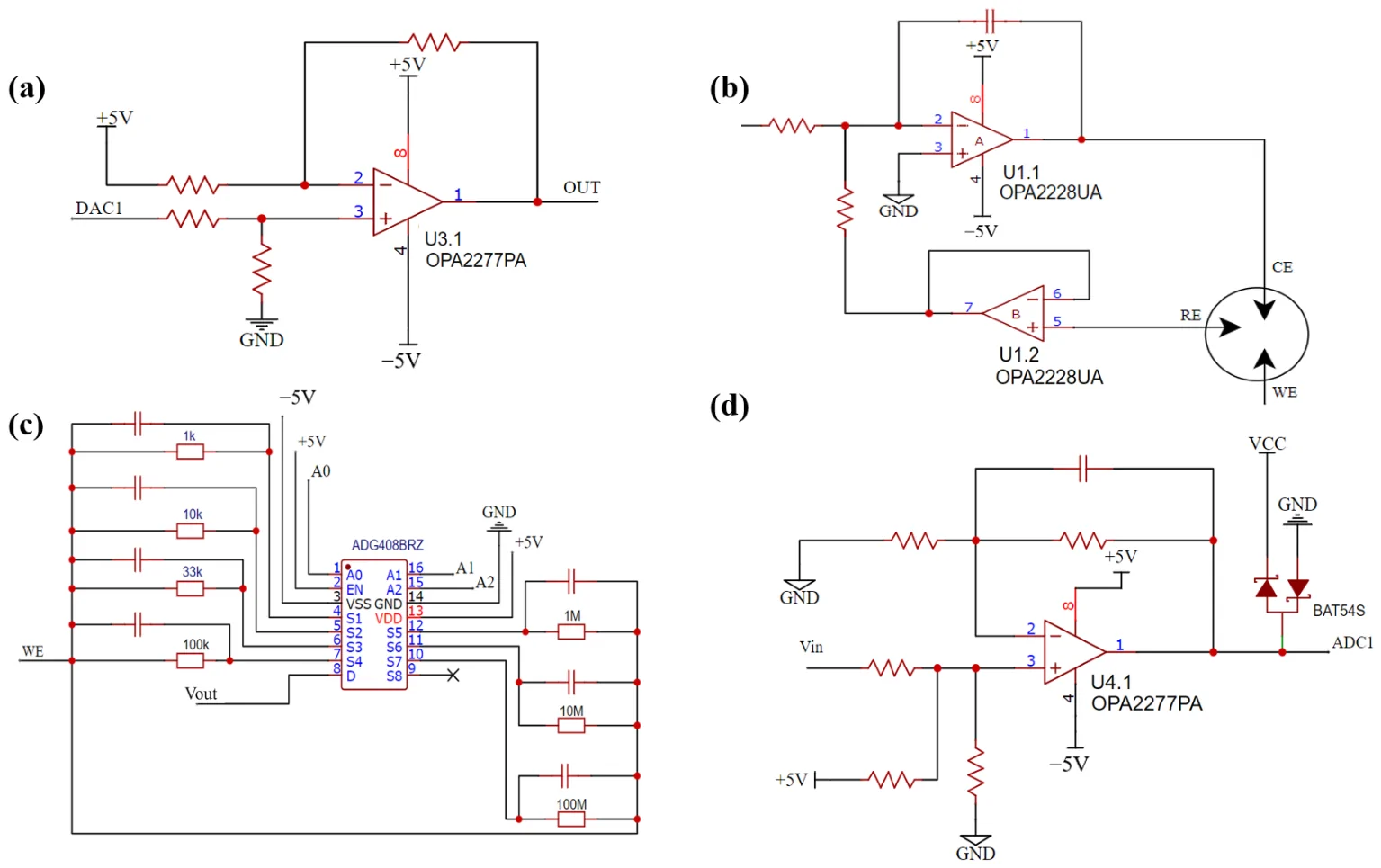
Main circuits of the detection device: (**a**) pulse-conditioning circuit; (**b**) potentiostat and three-electrode cell; (**c**) microcurrent detection circuit; and (**d**) level-shifting circuit.

The pulse-conditioning circuit translated the digital-to-analog converter output to the −0.20 to 1.20 V analytical range (Figure 2a). The potentiostat maintained the working-to-reference-electrode potential while the counter electrode supplied current (Figure 2b). Weak working-electrode currents were converted to voltage by the transimpedance stage, and the ADG408BRZ selected the feedback resistor (Figure 2c). A level-shifting stage placed the signal within the 0–3.3 V analog-to-digital converter range (Figure 2d). These circuits acquire the composite oxidation response of the extract; they do not confer molecular specificity.

#### 2.3.4. System Software Design

The analysis interface was developed in Python 3.10 with Qt 5.10.1 and provides instrument configuration, data reception, real-time curve display, quality-control prompts, model prediction, and data storage. Two computation modes use the same locked preprocessing and prediction pipeline. For field or mobile operation, the controller sends the 342-point vector by Bluetooth or Wi-Fi to a mobile gateway, which performs data-quality checking and accesses the cloud-hosted model through a 4G or Wi-Fi connection. For fixed factory or laboratory operation, preprocessing and the trained model run locally on a laptop or desktop computer. The predicted gLMS score, quality-control status, and curve are displayed automatically in either mode, so a laptop is optional in the field and manual interpretation of the voltammogram is not required.

#### 2.3.5. End-to-End Operating Sequence

The complete operating sequence proceeds from programmed DPV waveform generation and electrochemical current acquisition to digitization, wireless or wired transfer, automated quality control, and model inference. Circuit-level signal conditioning is described in Section 2.3.3 and software deployment in Section 2.3.4; the present subsection therefore defines only the end-to-end analytical sequence. Because the complete extract is analyzed without chromatographic separation, the recorded fingerprint represents the combined contribution of alkylamides and other electroactive matrix constituents.

### 2.4. System Evaluation and Validation

#### 2.4.1. Background Noise Measurement

**Figure 3:**
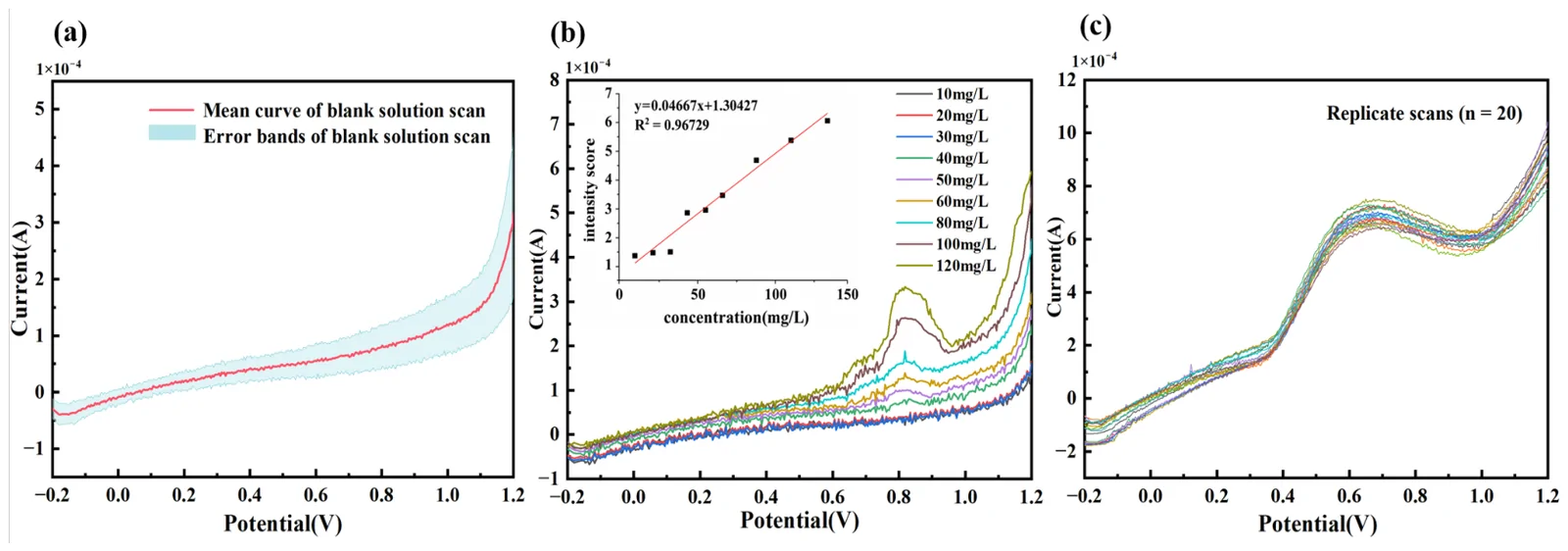
Analytical performance of the portable analyzer: (**a**) mean and variation envelope for ten blank measurements; (**b**) DPV responses and calibration of hydroxy-α-sanshool standards (10–120 mg L^−1^; y = 0.04667x + 1.30427, R^2^ = 0.96729); and (**c**) twenty scans of a Wenchuan red Szechuan pepper extract.

Ten independently recorded blank curves from a 1:1 mixture of 5 mL ethanol and 5 mL PBS were used to characterize instrumental background magnitude and variability (Figure 3a). The red line represents the blank mean and the blue envelope represents variation among the ten curves. These measurements also supplied the blank-response variability used for the instrumental limit of detection (LOD) and limit of quantification (LOQ) calculations. Baseline handling for pepper curves is described separately in Section 2.7.1; no blank curve was used as a fitted baseline function.

#### 2.4.2. Hydroxy-α-Sanshool Calibration, LOD, and LOQ

Nine hydroxy-α-sanshool levels (10, 20, 30, 40, 50, 60, 80, 100, and 120 mg L^−1^) were measured with the custom-built portable analyzer (Figure 3b). The concentration/response-index pairs were 10/2.077, 20/2.135, 30/2.047, 40/3.515, 50/3.609, 60/4.033, 80/5.358, 100/6.060, and 120/6.707. Linear regression gave y = 0.04667x + 1.30427 (R^2^ = 0.96729), where x is hydroxy-α-sanshool concentration (mg L^−1^) and y is the dimensionless electrochemical response index. The standard deviation of the ten independently measured blank response indices was 0.0402. Using LOD = 3.3σ/S and LOQ = 10σ/S, where σ is the blank-response standard deviation and S is the calibration slope, produced instrumental LOD and LOQ values of 2.84 and 8.61 mg L^−1^, respectively. Both values were below the lowest tested standard concentration of 10 mg L^−1^.

#### 2.4.3. Short-Term Repeatability

Twenty scans of the same Wenchuan red-pepper extract were recorded on the portable analyzer (Figure 3c). Across the 190 pairwise comparisons among these scans, the mean Pearson correlation was r = 0.9950 (range 0.9726–1.0000). This analysis assessed short-term conservation of fingerprint shape and potential position under repeated operation. These results indicate good overall repeatability under the standardized electrode treatment and measurement procedure.

### 2.5. Collection of Electrochemical Data

Immediately before electrochemical measurement, 15 mL of each ethanol extract was mixed with 15 mL of 0.10 M PBS (pH 5.6), giving a 1:1 (*v*/*v*) extract/PBS mixture and a final measurement volume of 30 mL. PBS served as the supporting electrolyte required for stable DPV acquisition; the pepper extracts were therefore not measured in pure ethanol.

For construction of the modeling dataset, the glassy-carbon working electrode was polished with 0.5 μm alumina slurry before each technical scan, rinsed with ultrapure water, and sonicated for 1 min to remove residual alumina [31,32]. The electrode was then repositioned in the fixed three-electrode holder. This standardized surface-renewal procedure was used to reduce potential carryover and electrode-fouling effects during calibration-dataset construction.

Fifteen technical scans were recorded for each of the 22 origin groups, yielding 330 DPV curves (225 red and 105 green). A curve was retained only if all 342 potential–current pairs were present, no value was missing or non-finite, the selected current channel showed no saturation, and the Pearson correlation with the pointwise median of the 15 curves from that origin exceeded 0.95. All 330 curves satisfied these predefined criteria; therefore, 0 curves and 0 origin groups were excluded.

**Figure 4:**
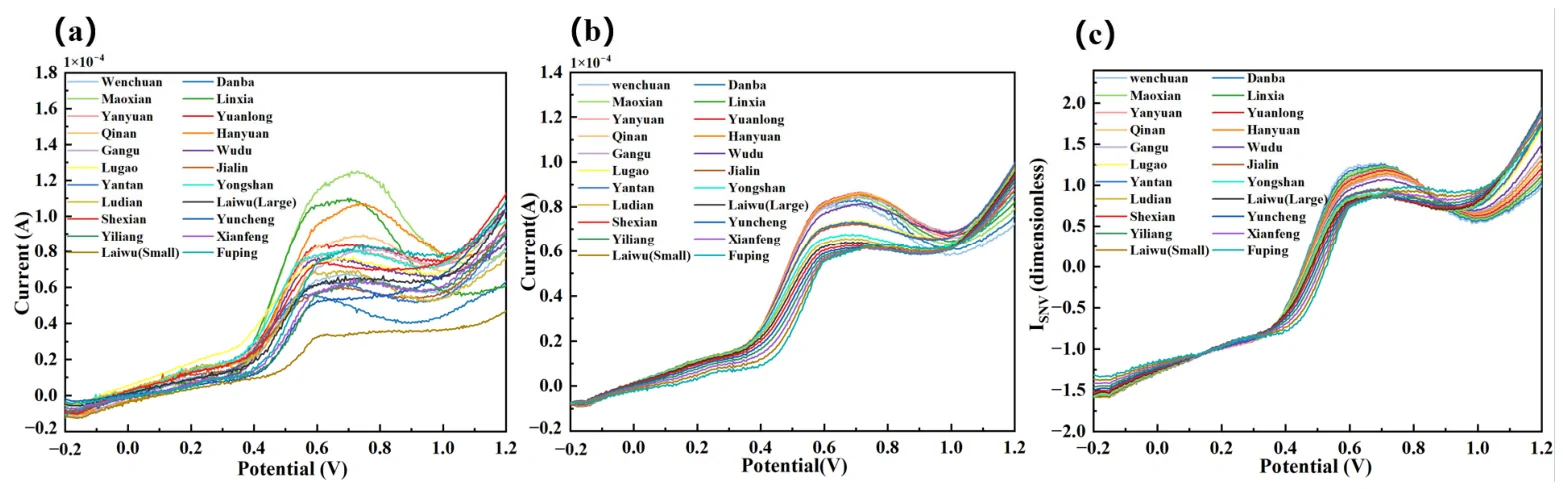
Preprocessing of one representative measured DPV curve from each of 22 origin groups: (**a**) raw current (A); (**b**) 11-point, third-order SG-smoothed current (A); and (**c**) SG followed by SNV transformation (dimensionless response).

For visualization, Figure 4 show one objectively selected measured curve per origin to prevent severe overlap and an unreadable 330-curve display. Within each origin, pairwise RMSE values were calculated among the 15 accepted SG-smoothed curves over all 342 potential points. The curve having the smallest mean RMSE to the other 14 curves—the within-origin medoid—was selected. The same measured curve was used for the raw, SG-smoothed, and SG–SNV panels in Figure 4. Representative-curve selection affected visualization only; all 330 accepted curves were retained for data partitioning, model training, cross-validation, and test evaluation.

A complete sample-to-result workflow required approximately 35–40 min, including standardized extraction. For a prepared extract and prepared electrode, DPV acquisition, automated quality control, model inference, and result display required less than 3 min. Routine operation used preset acquisition parameters and a standardized operating procedure; specialist support was reserved for calibration, maintenance, and troubleshooting.

### 2.6. Sensory Analysis

Sensory reference data were obtained through the Chinese National Institute of Standardization and the Sensory Evaluation Group of Jiangsu University. The trained assessor pool comprised 21 members, all of whom provided informed consent. A balanced incomplete-block design was used to control fatigue: 15 assessors evaluated each origin in each of three sessions, producing 45 ratings per origin. The study included 22 geographical origin groups; this number does not refer to the number of assessors. Presentation order was randomized within each session, and a minimum 5 min recovery interval was used between samples. Numbing intensity was recorded with the gLMS [33,34,35,36].

Training references were prepared from Szechuan pepper essential oil in ethanol and corresponded to gLMS anchors of 1.38 (barely detectable), 5.75 (weak), 16.22 (moderate), 33.11 (strong), 50.12 (very strong), and 95.5 (strongest imaginable sensation). The 45 ratings for each origin were averaged to obtain the reference value assigned to its 15 electrochemical scans.

### 2.7. Data Analysis and Model Development

#### 2.7.1. Data Preprocessing and Baseline Treatment

Each 342-point DPV vector was smoothed with an 11-point Savitzky–Golay (SG) filter using a third-order polynomial and a zero-order derivative. Standard normal variate (SNV) normalization was then applied independently to each SG-smoothed curve [37,38,39]:

$$x_{ij}^{SNV}=\frac{x_{ij}-{\bar{x}}_{i}}{s_{i}},\;\;s_{i}=\sqrt{\frac{\sum_{j=1}^{p}{\left(x_{ij}-{\bar{x}}_{i}\right)}^{2}}{p-1}},\;\;p=342$$

Here, x*_ij_* is the SG-smoothed current at potential point *j* of curve *i*, and ${\bar{x}}_{i}$ and s*_i_* are the mean and sample standard deviation of curve *i*, respectively. Mean centering removes the curve-wise constant offset, while division by s*_i_* reduces multiplicative scale differences. No independent polynomial, asymmetric least-squares, or other fitted-baseline correction was applied, and the SNV-transformed ordinate is dimensionless. Instrumental blank characterization in Figure 3a, SG smoothing, SNV normalization, and fitted-baseline correction therefore represent distinct operations.

#### 2.7.2. Dataset Construction and Partitioning

The modeling dataset comprised all 330 accepted curves from 22 origin groups: 225 red-pepper curves and 105 green-pepper curves. Red and green data were modeled separately. Kennard–Stone (K–S) sampling, rather than random selection, was used to form the 70:30 calibration/test partitions. Euclidean distances were calculated in the standardized 342-variable SG–SNV predictor space; after initialization with the most distant pair, the object having the largest minimum distance to the selected set was added iteratively until the calibration set was complete. Sensory gLMS labels were excluded from partition construction so that test-object selection remained independent of the response variable.

The resulting partitions contained 158 calibration and 67 test curves for red pepper and 74 calibration and 31 test curves for green pepper. Hyperparameter selection was performed only within the calibration partition using three-fold cross-validation. Random seed 42 was used only for stochastic model initialization and resampling. The reported test metrics therefore describe internal held-out performance within the sampled origin domain rather than external validation on newly collected origins.

#### 2.7.3. Feature Engineering, Model Selection, and Interpretation

CARS was applied only within each calibration partition using 50 Monte Carlo runs, an 80% sample draw per run, exponentially decreasing variable retention, adaptive reweighted sampling, and ten-fold internal cross-validation [40,41,42]. The variable subset at the minimum root mean square error of cross-validation (RMSECV), reached near run 35, was retained. Adjacent retained variables on the original potential grid were consolidated into contiguous potential windows to improve visualization and electrochemical interpretation. All transformations estimated from the calibration data were applied unchanged to the test set.

The mean SNV response in each of the three CARS windows was combined with six descriptors calculated from the SG-smoothed current curve: peak current (maximum current over −0.20 to 1.20 V), peak potential, endpoint current at 1.20 V, high-potential slope from an ordinary-least-squares fit over 0.80–1.20 V, trapezoidal integrated current over −0.20–1.20 V, and baseline-region mean current over −0.20–0.00 V. These nine features represent localized response, oxidation magnitude, peak position, integrated response, background level, and curve shape. Features were standardized using calibration-set means and standard deviations.

The same ten nonlinear candidates were evaluated for both pepper types: support vector regression (SVR), K-nearest neighbors (KNN), extreme gradient boosting (XGB), artificial neural network (ANN), one-dimensional convolutional neural network (1D-CNN), gradient-boosted decision trees (GBDT), PCA–SVR, PCA–ANN, PCA–GBDT, and PCA–1D-CNN [43,44,45,46,47]. Partial least squares regression (PLSR) and Elastic Net were added as linear baselines. Every algorithm used the same pepper-specific nine-feature matrix and K–S partition, and all hyperparameters were selected within the calibration partition.

PLSR searched 1–15 latent variables and selected 6 components for red pepper and 5 for green pepper. Elastic Net searched α from 0.001 to 10 on a logarithmic grid and l1 ratios of 0.1, 0.3, 0.5, 0.7, and 0.9; selected settings were α = 0.10 and l1 ratio = 0.50 for red pepper and α = 0.20 and l1 ratio = 0.50 for green pepper. Nonlinear models were tuned by 50 Bayesian-optimization iterations minimizing three-fold RMSE. Final settings were radial-basis-function SVR, C = 32, γ = 0.125, and ε = 0.10; distance-weighted KNN, k = 5 with Euclidean distance; XGB, 300 trees, maximum depth 4, learning rate 0.03, subsample 0.80, column subsample 0.80, and L2 regularization 1.0; ANN, input–128–64–1 architecture, ReLU activation, dropout 0.20, Adam learning rate 0.001, batch size 32, and mean-squared-error loss; 1D-CNN, 32 filters/kernel 5, max-pooling 2, 64 filters/kernel 3, global-average pooling, dense 32, dropout 0.20, and Adam learning rate 0.001; and GBDT, 200 trees, learning rate 0.05, maximum depth 3, and minimum leaf size 2. ANN and 1D-CNN were trained for at most 300 epochs with early stopping after 30 epochs without validation improvement. PCA-coupled regressors retained components explaining at least 99% of calibration-set variance.

Model performance was summarized by the coefficient of determination (R^2^) and RMSE [48,49]. RMSEC, RMSECV, and RMSET denote RMSE calculated for the training, cross-validation, and held-out test predictions, respectively; all are expressed in gLMS units:

Exploratory principal component analysis (PCA) was performed on the 22 origin-mean SG–SNV fingerprints, with each origin mean calculated from its 15 accepted curves. Model-agnostic permutation SHAP values were estimated for the fitted nine-feature pipelines using 300 feature-order permutations and the calibration-set median feature vector as the background. Mean absolute SHAP contributions were normalized to 100% within each pepper model. For green PCA–SVR, SHAP was applied to the complete pipeline, including PCA, so that contributions were returned for the original nine descriptors. Calculations used Python 3.10, scikit-learn 1.4, XGBoost 2.0, TensorFlow 2.15, and SHAP 0.45.

## 3. Results and Discussion

### 3.1. Preprocessing and Analytical Performance

Figure 4 compares one objectively selected representative DPV curve from each of the 22 origin groups before and after preprocessing. The raw representatives retain origin-dependent differences in current magnitude and curve shape [20,50,51]. The 11-point, third-order SG filter suppresses high-frequency noise without changing the 342-point potential grid, while curve-wise SNV corrects constant offset and multiplicative scale differences. The resulting dimensionless profiles preserve the broad potential-dependent response used for sensory prediction.

The ten blank curves in Figure 3a characterize background variability, whereas the standard-solution series in Figure 3b characterizes concentration-dependent instrumental response. The nine-level calibration yielded R^2^ = 0.96729 over 10–120 mg L^−1^. Based on the blank-response variability and calibration slope, the estimated LOD and LOQ were 2.84 and 8.61 mg L^−1^, respectively; thus, the lowest calibration standard (10 mg L^−1^) was above both estimated thresholds. These solution-phase limits are expressed in mg L^−1^ and are analytically distinct from the gLMS prediction RMSE discussed in Section 3.3.1 and Section 3.3.2.

For comparison, the optimized benchtop DPV method of Sun et al. [15] reported a linear range of 0.25–78.9 mg L^−1^ and an LOD of 0.03 mg L^−1^ for hydroxy-α-sanshool. The benchtop method provides greater trace-level sensitivity, whereas the present portable system extends the upper tested calibration concentration to 120 mg L^−1^ and prioritizes compact hardware and automated whole-extract prediction. Sugai et al. [52] reported a mean human detection threshold of 3.8 × 10^−5^ g mL^−1^ (38 mg L^−1^) for hydroxy-α-sanshool. The present standard-solution LOD, LOQ, and lowest tested level were below that sensory threshold. Figure 3c additionally shows high short-term fingerprint repeatability (mean pairwise r = 0.9950).

### 3.2. Sensory Evaluation of Numbing Intensity

**Figure 5:**
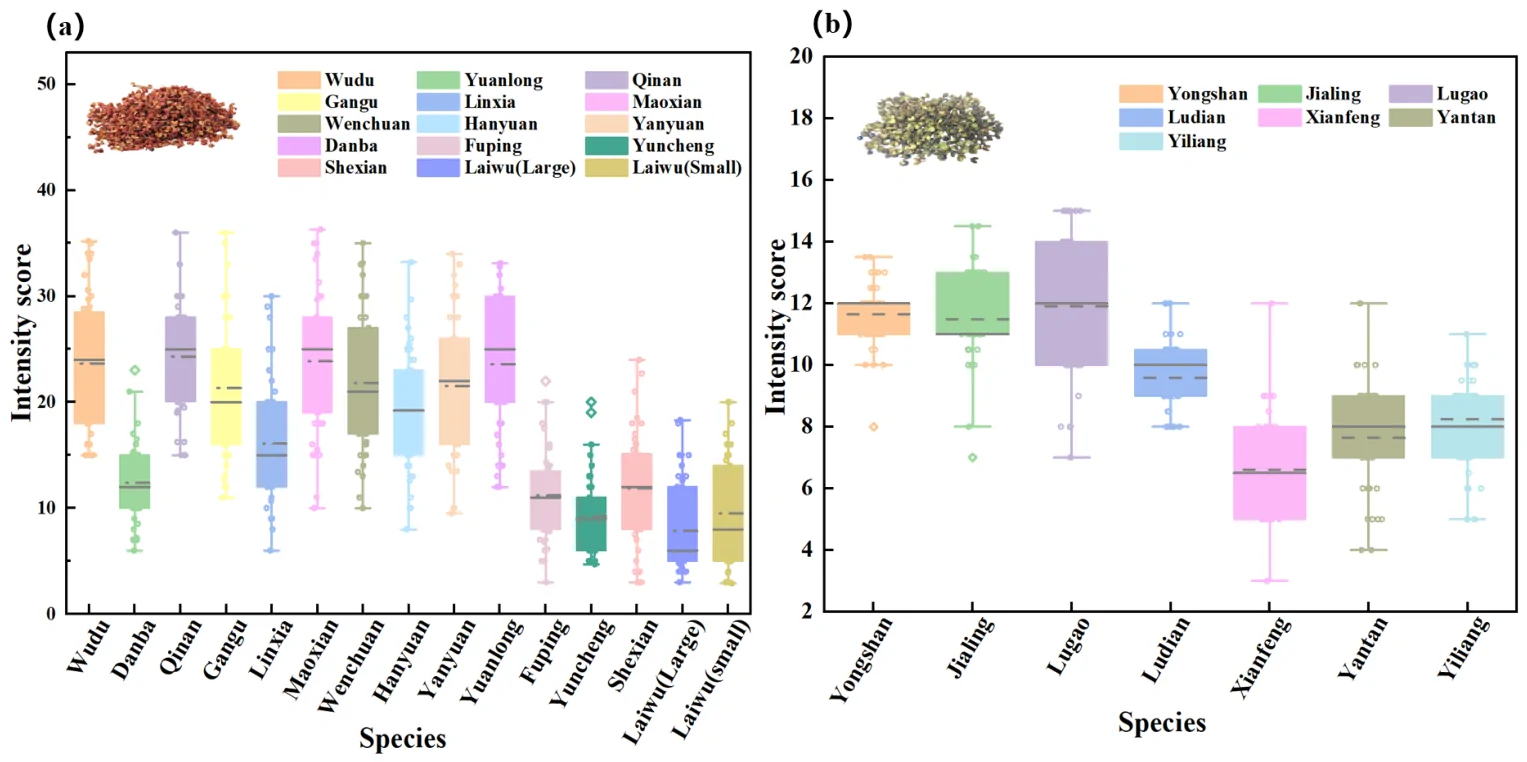
Sensory numbing-intensity distributions based on 45 ratings per origin: (**a**) 15 red Szechuan pepper groups and (**b**) 7 green Szechuan pepper groups. Boxes show the interquartile range, solid center lines show medians, dashed lines show means, and diamonds indicate outliers.

The sensory dataset contained 45 gLMS ratings for each of the 15 red and 7 green origins, generated in three sessions with 15 assessors per origin from the 21-member trained pool (Figure 5). The anchors of 1.38, 5.75, 16.22, and 33.11 correspond to barely detectable, weak, moderate, and strong intensity, followed by 50.12 (very strong) and 95.5 (strongest imaginable). Thus, Figure 5 describes perceived numbing magnitude rather than hydroxy-sanshool concentration.

Across the 675 red-pepper ratings, the mean was 17.16 ± 8.10 gLMS units (range 2.9–36.3), close to the moderate anchor, and individual ratings extended into the strong range. Origin means ranged from 7.86 to 24.32 gLMS units. Qinan (24.32), Maoxian (23.84), Wudu (23.65), and Yuanlong (23.60) formed the highest-intensity group, whereas Laiwu (large granule; 7.86), Yuncheng (9.22), Laiwu (small granule; 9.49), and Fuping (11.19) were substantially lower. This weak-to-moderately strong spread within red pepper demonstrates that numbing intensity was not determined by botanical type alone.

Across the 315 green-pepper ratings, the mean was 9.59 ± 2.54 gLMS units (range 3.0–15.0), between the weak and moderate anchors, and origin means ranged from 6.61 to 11.90 gLMS units. Lugao (11.90), Yongshan (11.64), and Jialing (11.49) had the highest means, whereas Xianfeng (6.61) and Yantan (7.64) were lower. The red-pepper mean exceeded the green-pepper mean by 7.57 gLMS units and the red distribution was wider; nevertheless, higher-intensity green origins overlapped with several lower-intensity red origins. Botanical type therefore shifted the overall distribution, while geographical origin contributed additional within-type variability.

The type-related difference is consistent with reported differences in the alkylamide and volatile profiles of *Z. bungeanum* and *Z. schinifolium* [21]. The substantial within-type variation is also consistent with a study of 19 geographical *Z. bungeanum* samples that found wide differences in total alkylamide content and sensory intensity [53], as well as sensory–chemometric discrimination of geographical origin [22]. Temperature, wind speed, and precipitation have been identified as climatic variables associated with amide accumulation [54], supporting plausible contributions from climate, altitude, cultivar, and postharvest history [52,55]. Maturity was controlled as an inclusion criterion rather than treated as an experimental factor; all lots met type-specific commercial-maturity criteria. Accordingly, systematic variation in this dataset is interpreted primarily as type- and origin-associated.

### 3.3. Internal Comparison and Interpretation of Numbing-Intensity Models

Separate models were developed for red and green pepper because their sensory ranges and electrochemical fingerprints differed. Table 2 and Table 3 report training, three-fold cross-validation, and held-out technical-scan test performance. A single precision convention is used throughout: R^2^ is reported to three decimal places and RMSE to two decimal places in gLMS units. Table 4 compares linear baselines on the same nine-feature matrices and K–S partitions.

#### 3.3.1. Red Szechuan Pepper Numbing-Intensity Model

All ten nonlinear candidates were evaluated using the same SG–SNV-derived nine-feature matrix and K–S partition. ANN retained the strongest held-out test performance (R^2^ = 0.937; RMSE = 3.09 gLMS units), followed by PCA–1D-CNN (R^2^ = 0.923; RMSE = 3.42 gLMS units) and 1D-CNN (R^2^ = 0.919; RMSE = 3.50 gLMS units). The consistent candidate set therefore supports selection of ANN without an asymmetric comparison between pepper types.

The red-pepper ANN test RMSE of 3.09 gLMS units corresponds to 9.3% of the observed 2.9–36.3 gLMS span. This error magnitude supports rapid differentiation and ranking of low-, medium-, and high-intensity samples within the investigated range. The sensory prediction error has a different measurand and unit from the hydroxy-α-sanshool LOD/LOQ and should therefore be interpreted separately.

#### 3.3.2. Green Szechuan Pepper Numbing-Intensity Model

Among the ten candidates, ANN achieved the highest training R^2^ (0.977; RMSEC = 1.46 gLMS units), whereas 1D-CNN achieved training R^2^ = 0.972 with RMSEC = 1.59 gLMS units. In cross-validation, 1D-CNN showed the strongest performance (R^2^ = 0.810; RMSECV = 3.92 gLMS units). On the test set, PCA–SVR gave the best result (R^2^ = 0.860; RMSET = 4.46 gLMS units), closely followed by 1D-CNN (R^2^ = 0.860; RMSET = 4.46 gLMS units after rounding). Thus, although ANN provided the strongest training fit, 1D-CNN and PCA–SVR showed better generalization.

The green-pepper PCA–SVR test RMSE of 4.46 gLMS units corresponds to 37.2% of the observed 3.0–15.0 gLMS span. The model retained useful ranking ability within this narrower range, but its quantitative precision was lower than that of the red-pepper ANN. This difference is consistent with the narrower sensory distribution and the smaller number of independent green-pepper origins.

**Figure 6:**
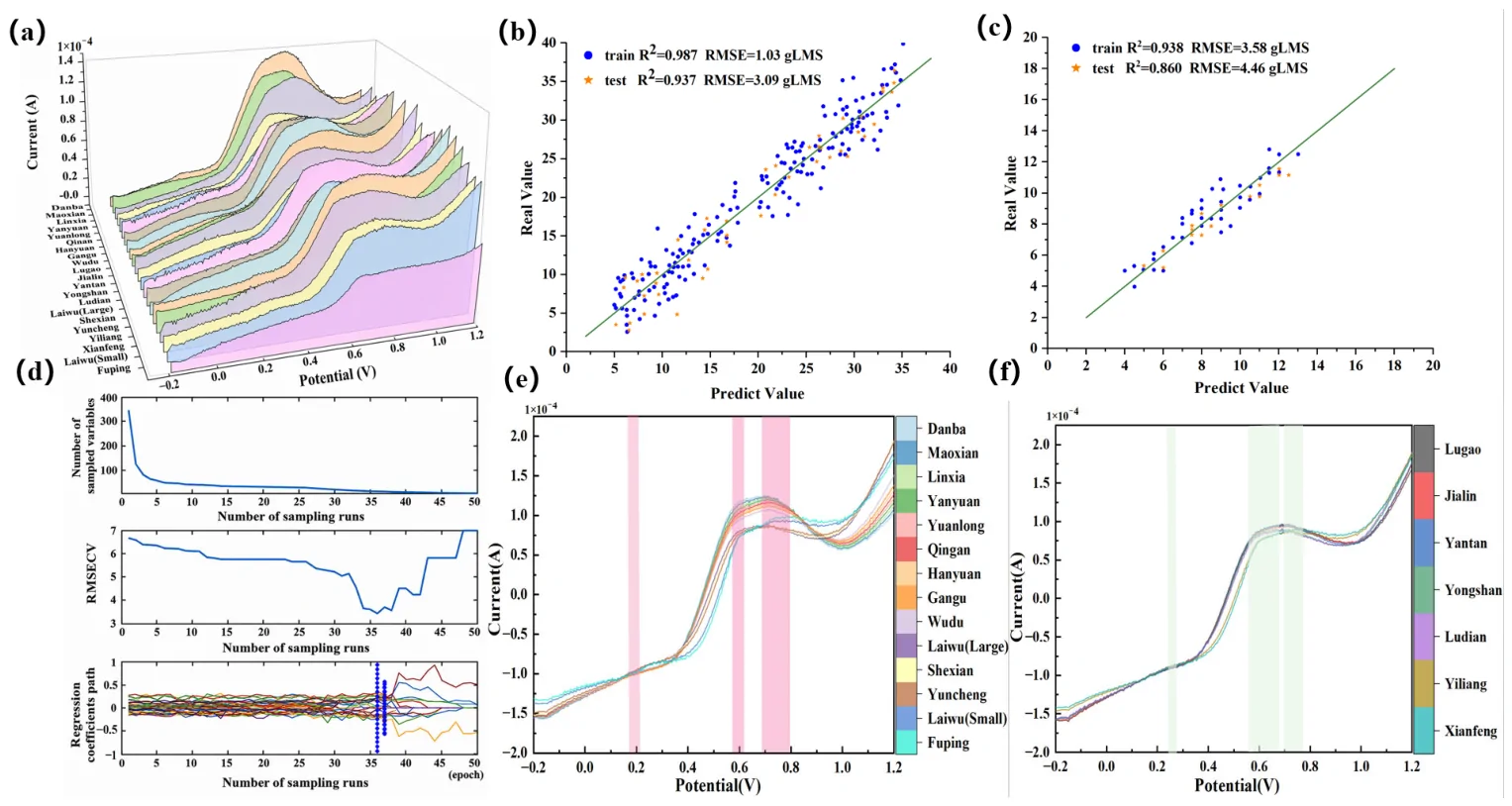
(**a**) Representative measured DPV voltammograms for the 22 origin groups; (**b**) measured versus predicted red-pepper scores for ANN (training: R^2^ = 0.987, RMSE = 1.03 gLMS units; test: R^2^ = 0.937, RMSE = 3.09 gLMS units); (**c**) measured versus predicted green-pepper scores for PCA–SVR (training: R^2^ = 0.938, RMSE = 3.58 gLMS units; test: R^2^ = 0.860, RMSE = 4.46 gLMS units); (**d**) enlarged CARS variable-selection trace; (**e**) enlarged red-pepper CARS windows at 0.17–0.21, 0.57–0.62, and 0.69–0.80 V; and (**f**) enlarged green-pepper CARS windows at 0.24–0.27, 0.56–0.68, and 0.69–0.77 V. Panels (**d**–**f**) are reproduced at full text width with the retained potential intervals marked on the original potential axis.

Figure 6a presents one representative measured DPV curve for each of the 22 origin groups, selected using the within-origin medoid criterion described in Section 2.5. Panels (b) and (c) distinguish training observations (blue circles) from held-out test observations (orange symbols) and report R^2^ and RMSE for both subsets. For red ANN, training/test R^2^ values were 0.987/0.937 and RMSE values were 1.03/3.09 gLMS units. For green PCA–SVR, the corresponding values were 0.938/0.860 and 3.58/4.46 gLMS units. The larger test errors than the training errors are consistent with evaluation on unseen technical scans.

#### 3.3.3. Feature Engineering and Mechanistic Interpretation

The 22 representative voltammograms in Figure 6a share a broad response increase from approximately 0.4 to 0.8 V, but peak magnitude, shoulder shape, and high-potential slope vary by pepper type and geographical origin. Because ethanol coextracts alkylamides, phenolic compounds, and other electroactive constituents, the complete profile is interpreted as a cross-reactive matrix fingerprint rather than a selective response from one molecule [56]. This distributed chemical response provides the mechanistic basis for multivariate rather than single-peak calibration.

During the 50 CARS Monte Carlo runs, the number of retained variables decreased rapidly under the exponentially decreasing function and adaptive reweighting progressively removed weak variables. Ten-fold RMSECV reached its minimum near run 35 before increasing (Figure 6d). For red pepper, the retained intervals were 0.17–0.21, 0.57–0.62, and 0.69–0.80 V (Figure 6e); for green pepper, they were 0.24–0.27, 0.56–0.68, and 0.69–0.77 V (Figure 6f). The intervals were selected from calibration-set predictive information and were not chosen manually from peak positions. The structured separation of preprocessing, feature representation, and variable selection follows the electrochemical feature-matrix strategy emphasized by Shukla et al. [57].

Both pepper types retained two neighboring intervals within approximately 0.56–0.80 V, coinciding with the shoulder and broad principal oxidation envelope. The hydroxy-α-sanshool standard produced a concentration-dependent response on the same platform, and previous DPV studies have demonstrated the electroactivity of hydroxyl-sanshools [15,20]. Szechuan pepper contains several structurally related conjugated unsaturated alkylamides, including hydroxy-α-, hydroxy-β-, and hydroxy-γ-sanshool. In an unseparated extract, their closely related structures are expected to generate partially overlapping responses rather than fully resolved compound-specific peaks. The middle- and high-potential CARS windows are therefore interpreted as different informative portions of an integrated hydroxyl-sanshool-related oxidation response, not as one-to-one assignments to individual isomers. Accordingly, these overlapping responses are treated as components of the integrated electrochemical fingerprint rather than as compound-specific positive or negative identification signals.

The lower-potential intervals at approximately 0.17–0.27 V are consistent with contributions from readily oxidizable phenolic constituents. These phenolic-related responses can influence the current magnitude and shape of the whole-extract DPV profile. For selective quantification of an individual hydroxyl-sanshool, overlapping phenolic responses would constitute analytical interference [58]. In the present study, however, the analytical endpoint is the sensory numbing intensity of the whole extract rather than the concentration of an individual sanshool. Phenolic-related responses are therefore retained together with sanshool-related responses as complementary components of the multivariate electrochemical fingerprint used for sensory prediction.

#### 3.3.4. PCA, Linear Baselines, and SHAP Interpretation

**Figure 7:**
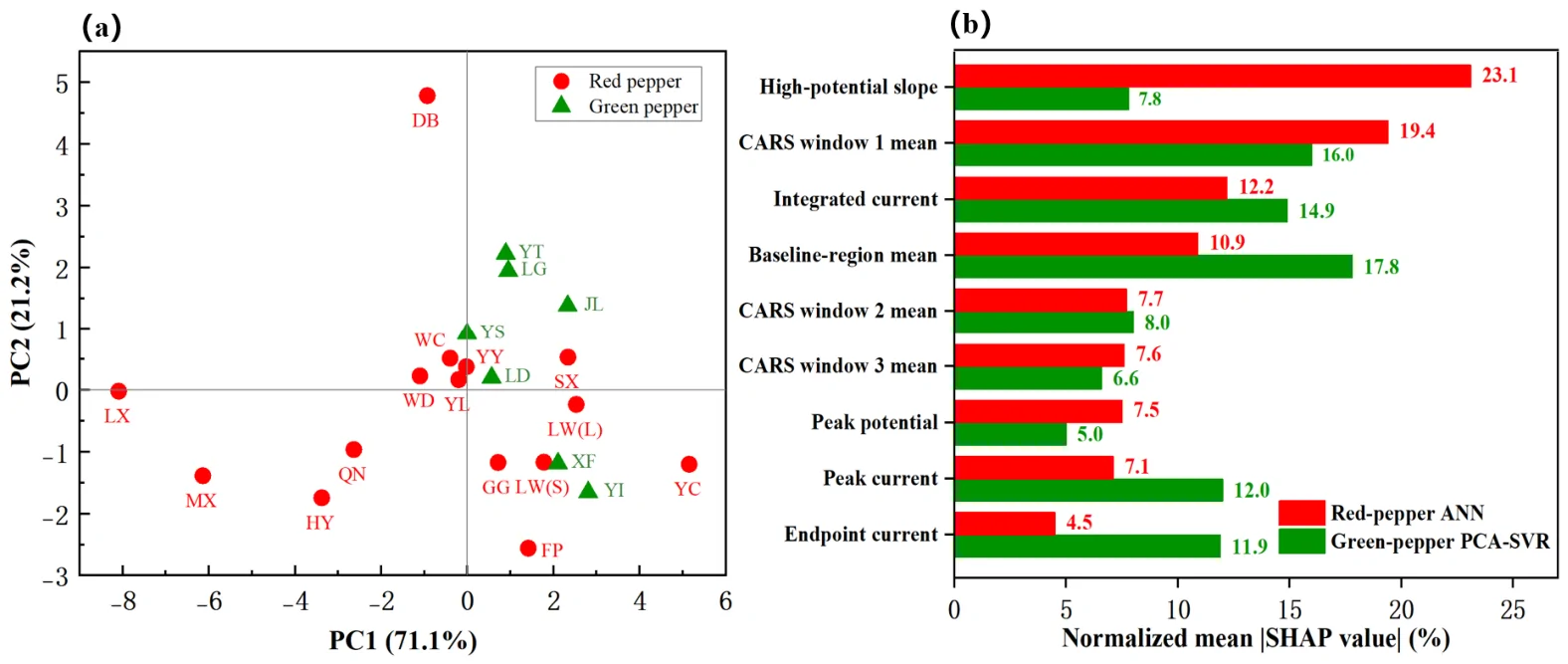
(**a**) PCA scores of the 22 origin-mean SG–SNV fingerprints (PC1 = 71.1%; PC2 = 21.2%). (**b**) Normalized mean absolute SHAP contributions (%) of nine electrochemical descriptors for the red-pepper ANN and green-pepper PCA–SVR.

PCA of the 22 origin-mean SG–SNV fingerprints explained 71.1% and 21.2% of variance in PC1 and PC2, respectively (92.4% cumulative; Figure 7a). Origin means were used only for this exploratory visualization so that geographical groups could be compared without overrepresenting the 15 technical scans from each origin; all 330 curves remained in regression modeling. Red and green origins showed partial separation with an overlapping central region. Red origins occupied a broader score space, with Danba separated mainly along positive PC2, Linxia and Maoxian toward negative PC1, and Fuping and Yuncheng occupying peripheral lower-PC2 and positive-PC1 positions, respectively. Green origins were comparatively concentrated in the central-to-positive PC1 region, although between-origin variation along PC2 remained evident. This broader dispersion of red-pepper fingerprints is consistent with the wider sensory distribution observed in Figure 5, where origin means ranged from 7.86 to 24.32 gLMS units for red pepper compared with 6.61 to 11.90 gLMS units for green pepper. The central overlap nevertheless indicates that the PCA should be interpreted as exploratory evidence of type- and origin-associated electrochemical variability rather than as complete botanical or geographical classification.

The linear baselines were weaker than the best nonlinear models on identical nine-feature matrices and K–S partitions (Table 4). For red pepper, six-component PLSR gave test R^2^ = 0.821 and RMSE = 5.20 gLMS units, and Elastic Net gave R^2^ = 0.845 and RMSE = 4.93 gLMS units, compared with ANN R^2^ = 0.937 and RMSE = 3.09 gLMS units. Relative to Elastic Net, ANN reduced RMSE by 37.3%. For green pepper, five-component PLSR gave R^2^ = 0.714 and RMSE = 6.51 gLMS units, and Elastic Net gave R^2^ = 0.742 and RMSE = 6.08 gLMS units, compared with PCA–SVR R^2^ = 0.860 and RMSE = 4.46 gLMS units. Relative to Elastic Net, PCA–SVR reduced RMSE by 26.6%. The nonlinear advantage was therefore retained on held-out observations rather than inferred from training fit alone.

SHAP analysis revealed distinct feature-contribution patterns between the red- and green-pepper models (Figure 7b). In the red-pepper ANN, the high-potential slope showed the largest normalized mean absolute SHAP contribution (23.1%), followed by CARS-window-1 mean (19.4%) and integrated current (12.2%), whereas peak current contributed 7.1%. This pattern indicates that red-pepper prediction depends more strongly on the evolution and shape of the high-potential response, localized potential-dependent information, and the overall electrochemical response than on the magnitude of a single oxidation peak. This interpretation is consistent with the red-pepper voltammograms in Figure 6a, where between-origin differences are expressed not only in current magnitude but also in shoulder structure and high-potential curve shape.

A different contribution pattern was observed for green pepper. In the PCA–SVR model, baseline-region mean (17.8%), CARS-window-1 mean (16.0%), integrated current (14.9%), peak current (12.0%), and endpoint current (11.9%) showed relatively distributed contributions, with no single descriptor accounting for a predominant share of the model attribution. Thus, the green-pepper model combines information from the low-potential response level, localized CARS-selected regions, overall integrated response, peak magnitude, and terminal high-potential response. Peak current remained informative in both models but did not dominate the prediction. Together with the CARS results in Section 3.3.3, these findings demonstrate that sensory numbing-intensity prediction depends on complementary information distributed across the DPV fingerprint rather than on a single peak-current measurement. CARS identifies where localized predictive information occurs, whereas SHAP clarifies how localized responses, signal magnitude, integrated response, and curve shape jointly contribute to the fitted prediction.

### 3.4. Technical and Operational Feasibility

In the present study, “portable” refers to the compact electrochemical analyzer rather than to integration of every sample-preparation step into one hand-held unit. The analyzer measures approximately 15 cm × 10 cm × 5 cm, weighs approximately 0.5 kg, and can operate for approximately 8 h from a 12 V rechargeable battery. Battery power, wireless transfer, and mobile/cloud inference permit field or mobile operation without a 220 V connection or laptop. At fixed industrial or laboratory sites, adapter power and local computer-based inference provide continuous operation without dependence on an external network. Both modes use the same locked SG–SNV preprocessing, quality-control rules, and trained models.

Sample preparation remains the largest component of the 35–40 min workflow. At a mobile testing station, the prescribed grinding, sonication, and centrifugation can be performed with independent commercially available rechargeable grinders, compact ultrasonic extractors, and DC-powered low-speed centrifuges; these auxiliary products are not components of the developed analyzer. At an industrial at-line station, multiple extracts can be prepared in parallel and then measured sequentially. For a prepared extract, DPV acquisition and automated prediction require less than 3 min. The system is therefore applicable to field screening, near-site analysis, and production-line-adjacent batch comparison rather than continuous in-line sensing of intact pepper.

The 15-scan protocol was used to construct and characterize the model-development dataset. Routine field analysis is intended to use one DPV acquisition per prepared sample and therefore requires one standardized electrode-polishing step before measurement. Preset parameters, automated data-quality checks, and direct score display reduce operator dependence; routine measurements can be performed after short hands-on training in standardized extraction, sample loading, and electrode care. Programmed potential-pulse cleaning in PBS will be investigated in future work as a potential strategy for simplifying electrode maintenance during repeated field measurements.

Although the dataset contains 330 curves, these measurements represent 22 geographical origin groups, including only seven green-pepper origins. Repeated scans strengthen measurement-repeatability assessment but do not replace independent population coverage. The current test results should therefore be interpreted as internal held-out performance within the sampled origin space. Additional origins, maturity stages, harvest seasons, production batches, and an independent external dataset are required to establish broader calibration transfer and industrial robustness.

## 4. Conclusions

This study established a portable battery-powered DPV fingerprinting system for rapid estimation of Szechuan pepper numbing intensity. All 330 curves from 22 origin groups passed predefined quality control and were retained. With a fully specified SG–SNV and feature-engineering workflow, ANN gave the strongest red-pepper test result (R^2^ = 0.937; RMSE = 3.09 gLMS units), while PCA–SVR gave the strongest green-pepper result (R^2^ = 0.860; RMSE = 4.46 gLMS units); both outperformed PLSR and Elastic Net. CARS and SHAP linked predictions to distributed potential-window, current-intensity, integrated-response, and curve-shape information, supporting interpretation of the device as a cross-reactive whole-extract sensor. The hydroxy-α-sanshool calibration (10–120 mg L^−1^) gave y = 0.04667x + 1.30427 (R^2^ = 0.96729), with instrumental LOD and LOQ values of 2.84 and 8.61 mg L^−1^. The approximately 0.5 kg analyzer can use mobile/cloud inference under battery power in the field or adapter power and local computation at a fixed site. The complete workflow requires approximately 35–40 min, whereas acquisition and automated prediction require less than 3 min for a prepared extract. Broader validation across additional origins, maturity stages, seasons, and independent production batches will strengthen future calibration transfer.

## Figures and Tables

**Table 1:** Nominal sensitivity settings, feedback resistors, and current measurement ranges.

Sensitivity (A/V)	Feedback Resistor (Ω)	Measurement Current Range
1 × 10^−6^	100 K	−2~12 μA
1 × 10^−5^	33 K	−6~36 μA
1 × 10^−5^	10 K	−20~120 μA
1 × 10^−4^	1 K	−200~1200 μA

Notes: Sensitivity is the nominal current-to-voltage setting; the ranges are the corresponding hardware measurement ranges.

**Table 2:** Internal technical-scan performance of ten nonlinear models for red Szechuan pepper using SG–SNV preprocessing and the K–S 70:30 partition. RMSE values are in gLMS units.

Preprocessing Method	Sample Set Division	Method	Train		Cv		Test	
			R_C_^2^	RMSEC	R_v_^2^	RMSEV	R_t_^2^	RMSET
SG-SNV	K-S[7:3]	SVM	0.960	2.03	0.789	5.13	0.893	4.43
		KNN	0.989	0.74	0.823	4.99	0.762	4.98
		XGB	0.979	1.58	0.752	5.73	0.634	7.22
		ANN	0.987	1.03	0.892	4.28	0.937	3.09
		1DCNN	0.982	1.27	0.874	4.52	0.919	3.50
		GBDT	0.972	1.75	0.781	5.32	0.842	4.75
		PCA-SVR	0.951	2.25	0.836	4.80	0.901	4.14
		PCA-ANN	0.975	1.61	0.851	4.63	0.910	3.82
		PCA-GBDT	0.966	1.90	0.774	5.41	0.826	5.05
		PCA-1DCNN	0.978	1.45	0.866	4.44	0.923	3.42

**Table 3:** Internal technical-scan performance of ten nonlinear models for green Szechuan pepper using SG–SNV preprocessing and the K–S 70:30 partition. RMSE values are in gLMS units.

Preprocessing Method	Sample Set Division	Method	Train		Cv		Test	
			R_C_^2^	RMSEC	R_v_^2^	RMSEV	R_t_^2^	RMSET
SG-SNV	K-S[7:3]	SVM	0.940	2.93	0.709	6.53	0.613	8.03
		KNN	0.950	0.05	0.714	6.18	0.529	8.34
		XGB	0.959	2.38	0.609	7.43	0.344	10.52
		ANN	0.977	1.46	0.753	5.88	0.627	7.91
		1DCNN	0.972	1.59	0.810	3.92	0.860	4.46
		GBDT	0.938	3.58	0.722	6.00	0.749	6.08
		PCA-SVR	0.938	3.58	0.782	5.42	0.860	4.46
		PCA-ANN	0.923	4.24	0.769	5.89	0.794	5.67
		PCA-GBDT	0.968	1.88	0.711	6.19	0.748	6.18
		PCA-1DCNN	0.954	2.47	0.803	4.22	0.850	4.99

**Table 4:** Linear baseline performance using the same nine-feature matrices and K–S 70:30 partitions as the nonlinear models. RMSE values are in gLMS units.

Pepper	Model	Selected Setting	Test R^2^	Test RMSE
Red	PLSR	6 components	0.821	5.20
	Elastic Net	α = 0.10; l1 ratio = 0.50	0.845	4.93
Green	PLSR	5 components	0.714	6.51
	Elastic Net	α = 0.20; l1 ratio = 0.50	0.742	6.08

## Data Availability

The original contributions presented in this study are included in the article. Further inquiries can be directed to the corresponding authors.
